# Combination of AKT inhibitor ARQ 092 and sorafenib potentiates inhibition of tumor progression in cirrhotic rat model of hepatocellular carcinoma

**DOI:** 10.18632/oncotarget.24298

**Published:** 2018-01-23

**Authors:** Zuzana Macek Jilkova, Ayca Zeybek Kuyucu, Keerthi Kurma, Séyédéh Tayébéh Ahmad Pour, Gaël S. Roth, Giovanni Abbadessa, Yi Yu, Brian Schwartz, Nathalie Sturm, Patrice N. Marche, Pierre Hainaut, Thomas Decaens

**Affiliations:** ^1^ Université Grenoble-Alpes, Saint-Martin-d’Hères, France; ^2^ Institute for Advanced Biosciences, Research Center Inserm U1209/CNRS 5309/UGA, Grenoble, France; ^3^ Izmir Institute of Technology, Department of Bioengineering, Izmir, Turkey; ^4^ ArQule Inc., Woburn, MA, USA; ^5^ CHU-Grenoble Département d’Anatomie et de Cytologie Pathologiques, La Tronche, France; ^6^ CHU-Grenoble Clinique Universitaire d’Hépato-Gastroentérologie, Pôle Digidune, France

**Keywords:** liver cancer, combination treatment, fibrosis, DEN-induced model, AKT inhibitor

## Abstract

The prognosis of patients with advanced hepatocellular carcinoma (HCC) is very poor. The AKT pathway is activated in almost half of HCC cases and in addition, long term exposure to conventional drug treatment of HCC, sorafenib, often results in over-activation of AKT, leading to HCC resistance. Therefore, it is important to assess the safety and the efficacy of selective allosteric AKT inhibitor ARQ 092 (Miransertib) in combination with sorafenib.

Here, we demonstrated *in vitro* that the combination of ARQ 092 with sorafenib synergistically suppressed proliferation, promoted apoptosis, and reduced migration. To test the effect of the combination *in vivo*, rats with diethylnitrosamine-induced cirrhosis and fully developed HCC were randomized and treated with vehicle, sorafenib, ARQ 092 or the combination of ARQ 092 with sorafenib; (n=7/group) for 6 weeks. Tumor progression, size of tumors and the mean tumor number were significantly reduced by the combination treatment compared to the control or single treatments. This effect was associated with a significant increase in apoptotic response and reduction in proliferation and angiogenesis. Sirius red staining showed a decrease in liver fibrosis. Moreover, treatments improved immune response in blood and in tumor microenvironment.

Thus, the combination of ARQ 092 with sorafenib potentiates inhibition of tumor progression and gives the possibility of therapeutic improvement for patients with advanced HCC.

## INTRODUCTION

Liver cancer (mainly hepatocellular carcinoma (HCC)) is reported to be the fifth most common cancer with second highest mortality among all cancers in adult men [1]. Viral hepatitis, chronic alcohol consumption and non-alcoholic steatohepatitis are the major causes of chronic liver inflammation which finally leads to HCC development. HCC that is diagnosed at an advanced stage has a very poor prognosis, and sorafenib is the only approved drug available. The multikinase inhibitor sorafenib, originally developed as a Raf kinase inhibitor, targets the MAPK/ERK pathway but also the vascular endothelial growth factor receptors (VEGF-R) and the platelet-derived growth factor receptor (PDGF-R). Even though sorafenib is the first drug that significantly increases clinical outcome of advanced HCC, its efficacy is modest with a median overall survival of 10.7 months versus 7.9 months with placebo in the pivotal phase III trial [2]. Moreover, long-term exposure to sorafenib often results in reduced sensitivity of the tumor cells, leading to acquired resistance. Therefore, new therapeutic treatments of HCC with better efficacy are urgently needed.

Growing evidence indicates that the phosphatidylinositol-3 kinase (PI3K)/AKT/mTOR-pathway is activated in approximately 50% of patients with cirrhosis and HCC [3, 4]. Moreover, sorafenib has been demonstrated to activate the AKT pathway in HCC cells [5] and this overactivation is considered to be one of the mechanisms of resistance to sorafenib treatment [6].

The serine/threonine kinase AKT, also known as protein kinase B or PKB, has become a major focus of attention mainly because of its critical role in regulating diverse cellular functions including metabolism, growth, proliferation, survival, transcription and protein synthesis. Activated AKT is known to inhibit apoptosis through its ability to phosphorylate several targets, including BAD, FoxO transcription factors, Raf-1 and caspase-9, that are critical for cell survival [7]. Therefore, the combination of sorafenib with an AKT-inhibitor could represent a new therapeutic strategy which could improve anti-tumor efficacy and overcome sorafenib resistance in HCC.

Recently, the combination strategy of sorafenib with mTOR inhibitors in HCC has been shown to be toxic and ineffective. Specifically, mTOR1 inhibitor everolimus in combination with sorafenib failed to show significant survival benefits compared to sorafenib alone [8]. Moreover, the same drug failed to demonstrate survival benefit in second line after failure of sorafenib, compared to placebo in a randomized phase 3 trial without patient’s selection [9]. It is necessary to emphasize that everolimus, similarly as other mTOR inhibitors, affects the mTORC1 protein complex, and not the mTORC2. This leads to increased AKT phosphorylation via inhibition on the mTORC1 negative feedback loop, while maintaining the mTORC2 positive feedback to AKT [10].

In contrast with mTOR inhibitors, direct inhibition of AKT seems to be an effective and nontoxic strategy. In recent work, we have demonstrated anti-tumor efficacy of ARQ 092, a highly selective allosteric inhibitor [11] that suppresses pan-AKT activity by blocking its phosphorylation and by preventing the inactive form from localizing into plasma membrane which together leads to strong and specific downregulation of downstream targets of AKT. Such high specificity was missing in catalytic AKT inhibitors that have been previously developed [12]. In addition, it was recently demonstrated that AKT inhibitors may reverse the acquired resistance to sorafenib *in vitro* [13]. However, to our knowledge the effect of the combination therapy of sorafenib + highly specific AKT inhibitor was never tested on HCC *in vivo*.

Therefore, in this study we combined ARQ 092 with sorafenib to investigate whether this therapeutic strategy could provide an improvement in treatment of advanced HCC, without increased toxicity.

In order to identify specific adverse events that could be related to the background of cirrhosis, newly developed therapeutic strategies should be pre-clinically tested in a relevant animal model of HCC developed on a cirrhotic liver. One of the well-established models that at present best reproduces human cirrhosis is diethylnitrosamine (DEN)-injured rats [14]. Therefore, we used the DEN-induced cirrhotic rat model with HCC to test safety and anti-tumor efficacy of the combination of sorafenib with AKT-inhibitor ARQ 092 (Supplementary Figure 1).

## RESULTS

### Combination of sorafenib and ARQ 092 suppresses cell proliferation, promotes cell apoptosis, and reduces migration

**Figure 1:**
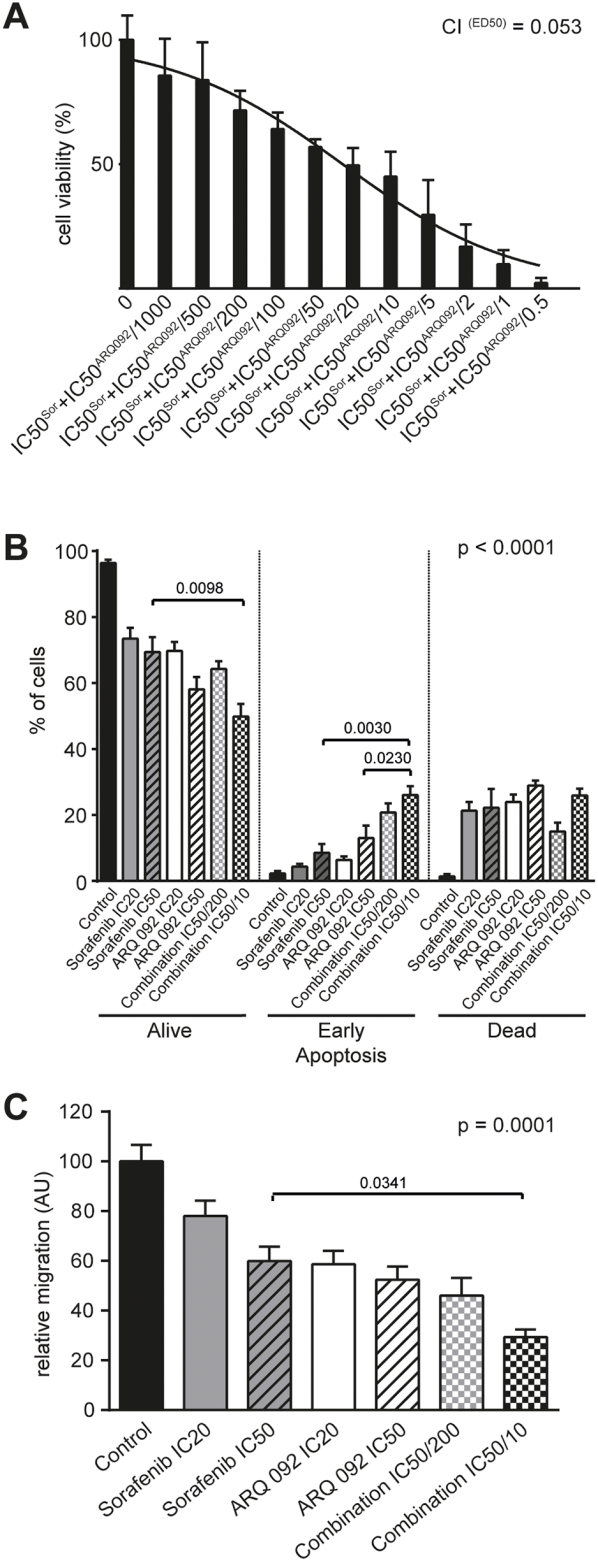
Effect of Combination treatment on Hep3B cell viability, apoptosis and cell migration **(A)** MTT assay on Hep3B cell line after 48h of treatments showing significant decrease in cell viability with increasing concentrations of combination treatment of ARQ 092 and sorafenib (constant ratio IC_50_: IC_50_). Combination index (CI = 0.053) at effective dose 50 (ED_50_) revealed strong synergy. **(B)** Additive effects of combination treatment of ARQ 092 and sorafenib on apoptosis in Hep3B after 48h of exposure. P values in graph represent ANOVA comparison of ARQ 092 IC_50_, sorafenib IC_50_ and Combination IC_50_/10. P value of ANOVA test of all groups is indicated in the corner of the graph (p <0.0001). **(C)** The quantification of migration (decrease of width of the wound after 24h) in Hep3B. Additive effects of combination treatment of ARQ 092 and sorafenib. P values in graph represent ANOVA comparison of ARQ 092 IC_50_, sorafenib IC_50_ and Combination I IC_50_/10. P value of ANOVA test of all groups is indicated in the corner of the graph (p =0.0001). Control was set as 100%, values are means ± SE from three independent experiments performed in triplicates (A) and in duplicates (B, C).

We previously determined the half maximal inhibitory concentration (IC_50_) of single treatment on each cell lines ([11], Supplementary Table 1). To determine the effect of the combination treatment on cell growth, we used a mixture of IC_50_ of single treatments (i.e. IC_50_^Sorafenib^+IC_50_^ARQ 092^). MTT assays showed a drastic decrease in proliferation rate for Hep3B (Figure 1A), HepG2, Huh-7 and PLC/PRF cell-lines (Supplementary Figure 2A). The calculated combination index (CI) values (for details see supporting information), revealed strong synergistic effect of the combination treatment of sorafenib and ARQ 092 on cell growth as summarised in the Supplementary Table 2. Combination IC_50_/200 (IC_50_^Sorafenib^+IC_50_^ARQ 092^/200) and Combination IC_50_/10 (IC_50_^Sorafenib^+IC_50_^ARQ 092^/10) were used for further experiments.

We observed a significant decrease in cell-viability in the combination and single treated groups in all tested cell lines in comparison to the control (p<0.0001), (Figure 1B and Supplementary Figure 3). Combination IC_50_/10 significantly increased early apoptotic cells in Hep3B compared to sorafenib IC_50_ (p=0.003) or ARQ 092 IC_50_ (p=0.023), (Figure 1B).

A wound-healing assay revealed that after 24h, the combination IC_50_/10 reduced migration of Hep3B significantly more than the sorafenib IC_50_ (Figure 1C, Supplementary Figure 4B). Moreover, in other cell lines, the migration of cells was decreased significantly in the combination IC_50_/10 treatment compared to both of the IC_50_ single treatments (Supplementary Figure 4A, 4B). Similar results were obtained when cell velocity was assessed by cell tracking with time-lapse microscopy showing that wound-healing slow was related to cell migration inhibition and not decrease of cell proliferation (Supplementary Figure 4C).

All together, these results demonstrate that the combination of sorafenib and ARQ 092 synergistically suppresses cell proliferation, promotes cell apoptosis, and in an additive manner reduces migration in all tested human cell-lines.

### Combination treatment in DEN-induced cirrhotic rat with HCC

To characterise the safety in a cirrhotic model and the anti-tumor efficacy of the combination of sorafenib and ARQ 092 in HCC, DEN-induced cirrhotic rats with HCC were treated during six weeks by sorafenib, ARQ 092, the combination of both drugs or the untreated control group, as specified in Supplementary Figure 1. In a previous study, the treatment schedule of ARQ 092 was 7 days on and 7 days off [11] but in this study, the schedule was changed to 5 days on and 9 days off in the ARQ 092 single treatment group and in the combination group to prevent possible side effects when combining with sorafenib treatment.

Safety data are summurized in Table 1. No significant differences in body weight were observed at the end of the treatment. The weight of the liver was lower in the ARQ 092 group compared to the control group, and in the combination group compared to the control and sorafenib group. Assessment of triglycerides in liver did not show any difference between groups (p=0.9743). Blood sample analysis revealed that none of treatments affect glucose, cholesterol or triglyceride blood concentrations. Similarly, kidney functions were not affected by treatments as plasmatic creatinine levels did not differ between groups. There was no statistical difference in transaminases, alkaline phosphatase (ALP) and prothrombin time among all groups. However, serum levels of AFP were significantly decreased by ARQ 092 and the combination treatment compared to the control group. We observed a significant decrease in total bilirubin and an increase in albumin level in ARQ 092 group compared to the control, and a decrease in GGT level in the combination group compared to the control. No difference was observed between the sorafenib and control groups.

**Table 1:** Clinical and biological analyses

		Control (n=7)	Sorafenib (n=7)	ARQ 092 (n=7)	Combination (n=7)	ANOVA p-values
**Body Weight (g)**		290±7.4	291±2.0	270±6.0	274±5.6	*ns*
**Liver**	Weight (g)	14.8±1.3	13.4±0.7	11.0±0.5^*^	10.9±0.5^**,#^	*0.0026*
	TG (g/L)	28.9±4.4	30.5±3.4	29.1±3.0	30.1±2.1	*ns*
**Blood**	Albumin (g/dL)	3.69±0.03	3.71±0.01	4.07±0.11^*,##^	3.76±0.05	*0.0046*
	AFP (ng/mL)	0.82±0.17	0.44±0.12	0.33±0.09^*^	0.27±0.04^*^	*0.0151*
	AST (U/L)	101.3±3.3	95.2±3.9	92.3±3.6	91.1±2.5	*ns*
	ALT (U/L)	73.1±5.2	73.0±3.9	67.6±6.3	68.4±2.2	*ns*
	ALP (U/L)	224±7.1	219±7.7	255±24.8	264±14.2	*ns*
	GGT (U/L)	21.5±3.8	15.9±2.8	13.3±1.9	7.4±1.4^**^	*0.0073*
	PT (s)	16.3±0.4	18.7±1.8	16.7±0.2	16.5±0.4	*ns*
	Total Bilirubin (mg/dL)	0.21±0.01	0.18±0.01	0.16±0.01^*^	0.17±0.01	*0.0211*
	Creatinine (mg/dL)	0.35±0.03	0.36±0.02	0.38±0.02	0.36±0.02	*ns*
	GLU (mg/dL)	128±3.4	142±4.3	153±4.7	142±4.8	*ns*
	Cholesterol (mg/dL)	86.6±6.5	84.9±4.0	102±7.3	88.7±5.1	*ns*
	TG (g/L)	62.2±11.5	75.1±11.2	60.2±7.7	67.7±10.1	*ns*

Abbreviations: AFP, alphafetoprotein; AST, aspartate aminotransferase; ALT, alanine aminotransferase; ALP, alkaline phosphatase; GGT, Gamma-glutamyl transpeptidase; PT, Prothrombin time. Values are means ± SE. Significant difference compared to control; ^*^: p<0.05; ^**^: p<0.01; ^***^: p<0.001; ^****^: p<0.0001. Significant difference between ARQ 092 and Sorafenib; ^##^: p<0.01.

Thus, our results showed that ARQ 092 and the combination treatment improve liver function but do not interfere with lipid or glucose metabolism (two major side effects of mTOR inhibitors).

**Figure 2:**
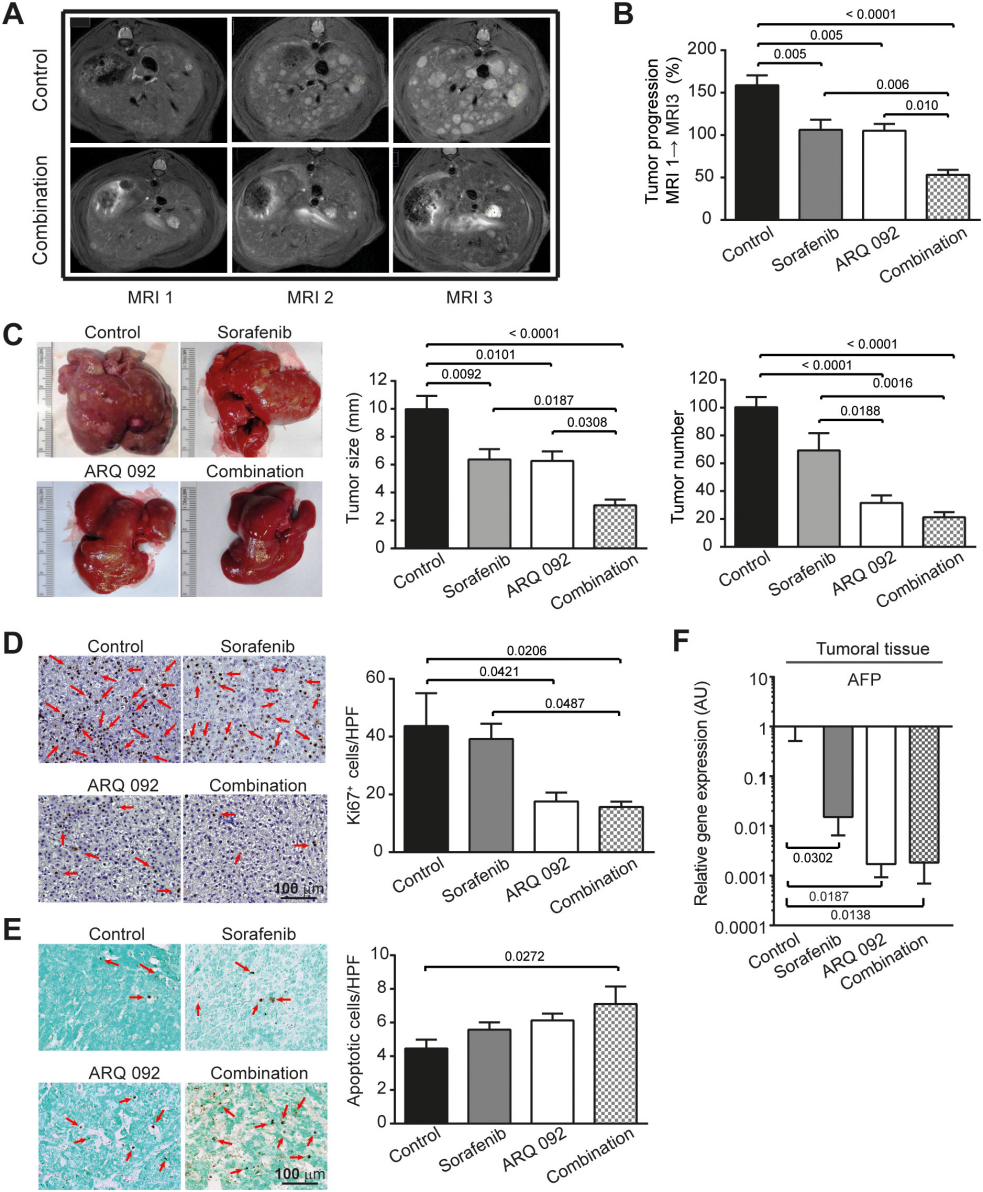
Anti-tumor effect of combination treatment **(A)** Representative pictures of abdominal MRI 1, 2 and 3 scan of non-treated rat and rat treated by combination treatment. **(B)** Tumor progression assessment by comparison of tumor size on MRI 1, 2 and 3 in Control, sorafenib, ARQ 092 and Combination group. **(C)** Macroscopic examination of livers with assessment of tumor size (middle panel) and tumor number at the surface of livers (right panel). **(D)** Representative images of nuclear Ki67 staining (arrow), 20x magnification with quantification of Ki67 staining per high power field (HPF). **(E)** Representative images of apoptosis induction (right panel) determined by TUNEL immunostaining (arrow), 20x magnification with quantification of apoptotic cells per HPF. **(F)** qPCR analysis of alpha fetoprotein (AFP) gene expression in tumor liver samples. The scale of the Y axes are Log 10, control was set as 1, values are means ± SE, n=7/group. Comparison of means was done by ANOVA test with Tukey correction.

The effect of ARQ 092, sorafenib and the combination of both agents on tumor progression was assessed by a liver MRI scan, Figure 2A. As illustrated in Figure 2B, tumor progression was significantly reduced by sorafenib (by 33.0±10.3%; p=0.005) and ARQ 092 (by 33.8±10.6%; p=0.005) compared to the control. The greatest decrease in tumor progression rate was observed in the combination group when compared with the control (66.6±10.6%; p<0.0001), indicating an additive effect of sorafenib and ARQ 092 on the control of tumor progression. Similarly, the combination treatment significantly reduced tumor progression compared to sorafenib (50.1±13.3%; p=0.006) and ARQ 092 (49.6±14.1%; p=0.010).

MRI analyses were further confirmed by macroscopic examination of the liver (Figure 2C), which revealed significantly smaller mean tumor size in the sorafenib (6.3 ± 0.8 mm), ARQ 092 (6.2 ± 0.8 mm) and combination group (3.0 ± 1.1 mm) compared to the control rats (9.9 ± 1.1 mm) with statistical significance p=0.0092, p=0.0101 and p<0.0001, respectively. Mean tumor size in the combination group was significantly reduced compared to single agents, sorafenib (p=0.0187) or ARQ 092 (p=0.0308), confirming that the combination treatment is superior to the single agents.

The macroscopic counting of tumors revealed a significantly lower number in rats treated by ARQ 092 and the combination group compared to the control and sorafenib groups. In fact, while the mean number of tumors on the liver surface of the control rats was 109.5±14.5, significant reduction was observed in the ARQ 092 treated rats (31.5±14.8; p<0.0001) and in the combination (21.21±14.5; p<0.0001). Similarly, the ARQ 092 and combination groups displayed a significantly lower number of tumors compared to tumor numbers (69.21±11.5) in the sorafenib-treated animals (p=0.0188 and p=0.0016 respectively), Figure 2C. Accordingly, the frequency of Ki67-positive nuclei was significantly reduced in ARQ 092 (p=0.0421) and in the combination group (p=0.0206) compared to the control group. The combination treatment also significantly reduced the Ki67 proliferation marker compared to sorafenib group (p=0.0487), Figure 2D. TUNEL immunostaining showed that only the combination group significantly induced apoptosis (p=0.0272), Figure 2E. The sorafenib treatment showed no statistical significance for Ki67 (89.8 ± 12.1 % of control, p=0.9563) or TUNEL (124.9 ± 9.6 % of control, p=0.4566), Figure 2D and 2E.

Real time qPCR analyses of alpha fetoprotein (AFP), HCC tumor marker, showed a reduced expression in all treated groups compared to the control (Figure 2F), with a much stronger effect in the combination group (p=0.0138).

Overall, we observed an additive effect of the combination of sorafenib and ARQ 092 on tumor progression and tumor size. Moreover, the combination significantly reduced tumor proliferation in DEN-induced HCC rat model, and was clearly more effective than sorafenib and/or ARQ 092 monotherapies.

### Effect of combination treatment on tumor vascularization and liver fibrosis

**Figure 3:**
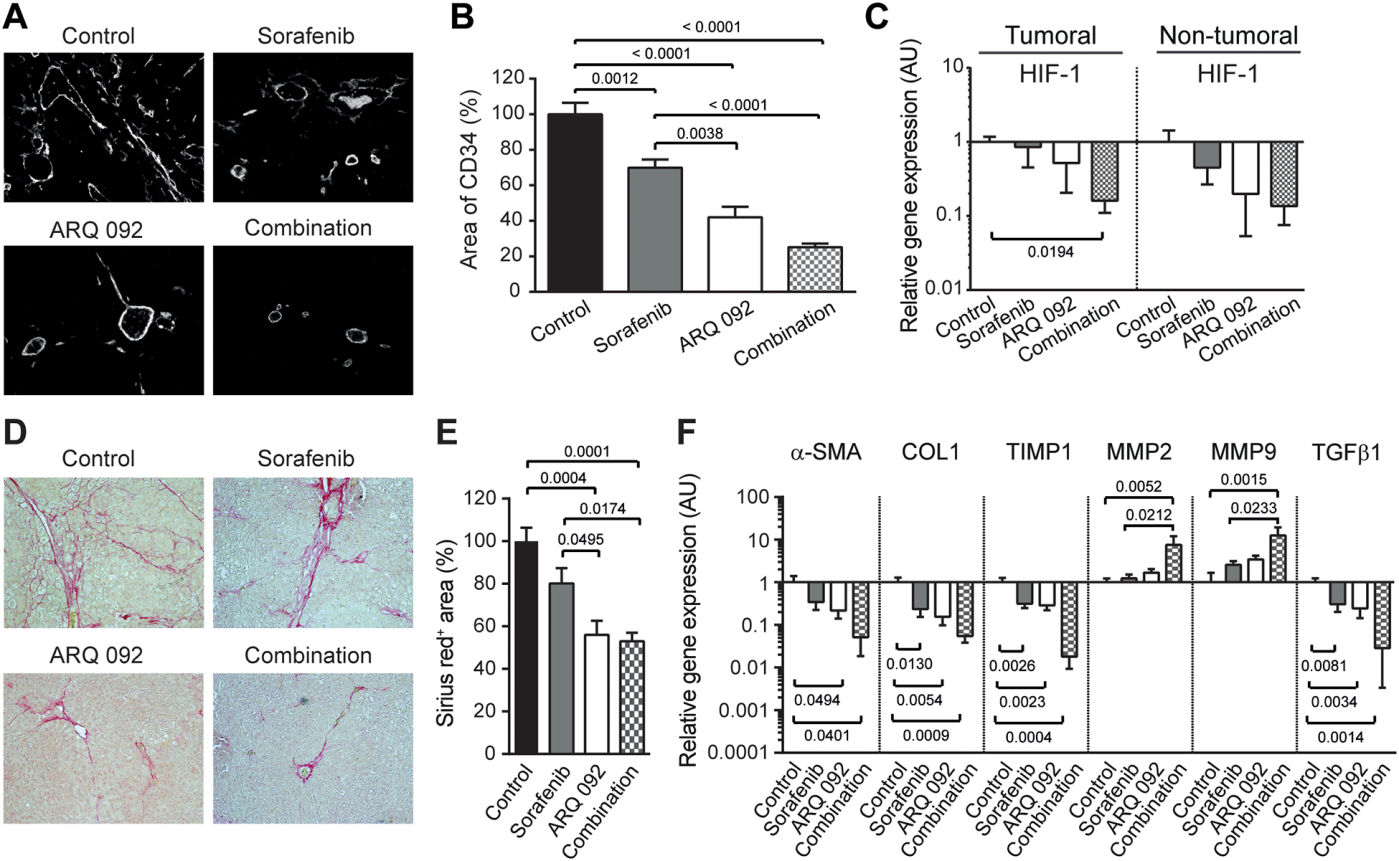
Effect of Combination treatment on tumor vascularization and liver fibrosis **(A)** Representative pictures of CD34 immunofluorescence staining of liver tissue. **(B)** Quantification of CD34 staining, control was set as 100, values are means ± SE. **(C)** qPCR analysis of Hypoxia-inducible factor (HIF)-1 gene expression in tumoral and non-tumoral liver samples. Control was set as 1. Values are means ± SE. N=7/group. Comparison of means was done by ANOVA test with Tukey correction. **(D)** Representative histological images of livers stained with Sirius red. **(E)** Quantification of Sirius red staining area per total area; control was set as 100 %. **(F)** qPCR analysis of alpha smooth muscle actin (α-SMA), Collagen (COL)1, TIMP Metallopeptidase Inhibitor 1 (TIMP1), Matrix metalloproteinase-2 (MMP2), Matrix metalloproteinase-9 (MMP9) and transforming growth factor (TGF)β gene expression in non-tumoral liver tissue. The scale of the Y axes are Log 10, control was set as 1. Values are means ± SE. N=7/group. Comparison of means was done by ANOVA test with Tukey correction.

Anti-angiogenic effect of treatment was determined by immunostaining of liver tissue, using a rat-specific anti-CD34 antibody. Major structural abnormalities of the vasculature were observed in control livers, and tissues from all treated groups demonstrated normalization of vasculature, Figure 3A. Similarly, the quantification of vascular density revealed that both sorafenib and ARQ 092 significantly decreased angiogenesis, but to smaller extent than the combination treatment. In fact, sorafenib decreased vascular density by 30% (p=0.0012), ARQ 092 by 58% (p<0.0001) and the combination by 75% (p<0.0001) compared to non-treated rats (Figure 3B). The gene expression of hypoxia-inducible factor (HIF-1), considered a marker of tumor hypoxia, tended to be decreased by all treatments but only the combination treatment significantly reduced HIF-1 expression in tumor tissue compared to the control (p=0.0194), Figure 3C.

Liver fibrosis was analyzed by Sirius red staining. As shown in Figure 3D and 3E, fibrotic tissues were significantly reduced in the ARQ 092 and combination groups compared to the control and sorafenib groups.

Improvement of liver fibrosis by ARQ 092 and the combination treatment was confirmed by qPCR analysis of non-tumoral tissue (Figure 3F). The expression of markers of liver fibrosis (alpha smooth muscle actin (α-SMA), collagen 1 and transforming growth factor β1 (TGFβ 1)) was significantly downregulated in non-tumor liver samples in ARQ 092 and the combination groups compared to the control group. Accordingly, the tissue inhibitor of metalloproteinases-1 (TIMP-1) was decreased by all treatments compared to the control whereas matrix metalloproteinases MMP2 and MMP9 were significantly upregulated in the combination group compared to the control and sorafenib groups. This effect on the matrix pathway was specific for non-tumor tissue. Thus, ARQ 092 and the combination treatment significantly decreased hepatic collagen deposition and improved liver fibrosis in DEN-induced cirrhotic rats, while sorafenib only had a mild effect.

### Effect of combination treatment on AKT and ERK pathway

Western blot analyses showed that ARQ 092 and the combination of ARQ 092 with sorafenib treatment blocked phosphorylation of AKT^(Ser473)^ in all human HCC cell lines at both IC_20_ and IC_50_ concentrations (Supplementary Figure 5).

**Figure 4:**
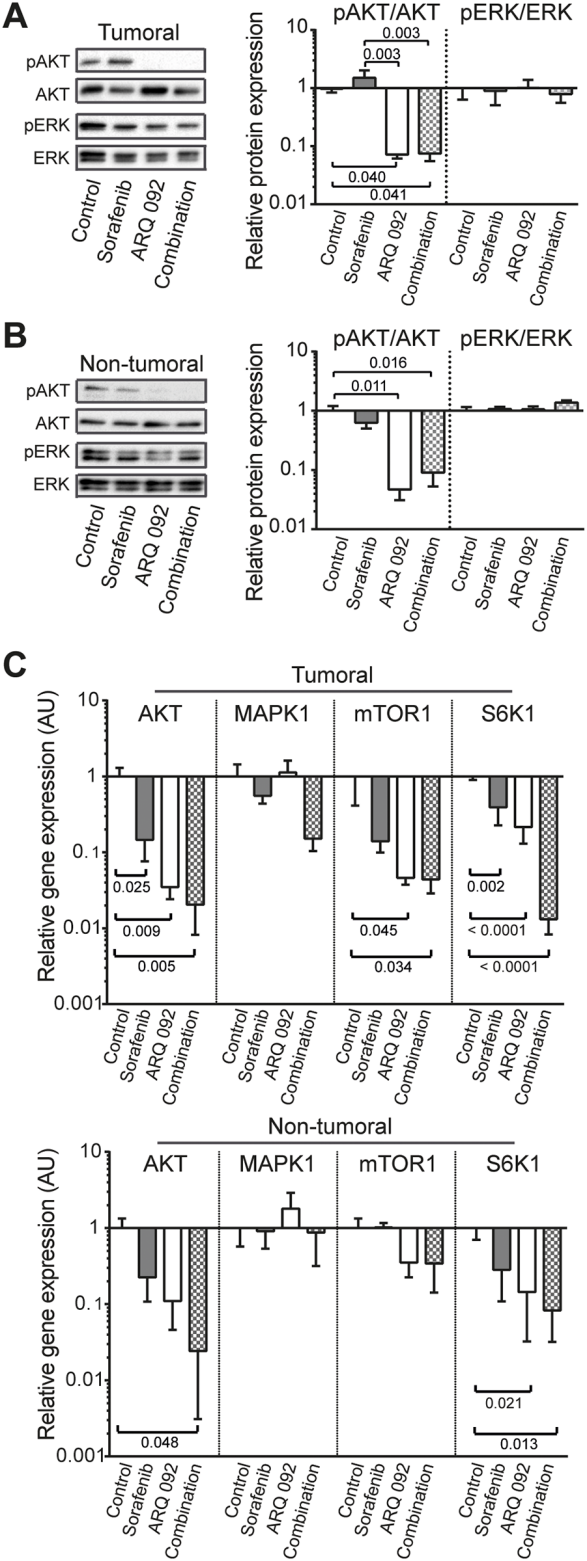
Effect of Combination treatment on AKT and ERK pathways Western blot analysis of pAKT/AKT and pERK/ERK in **(A)** tumoral and **(B)** non-tumoral liver tissue. pAKT and pERK were stained first and after development, the membranes were stripped followed by staining of AKT and ERK. **(C)** qPCR analysis of the expression of AKT, MAPK, mTOR, S6K1 in tumoral (upper panel) and non-tumoral (lower panel) liver tissue. The scale of the Y axes are Log 10, control was set as 1, values are means ± SE. N=7/group. Comparison of means was done by ANOVA test with Tukey correction.

In the *in vivo* model, ARQ 092 and the combination treatment strongly inhibited phosphorylation of AKT^(Ser473)^ in both tumor and non-tumor liver tissues (Figure 4A and 4B). qPCR analyses demonstrated a significant decrease in AKT gene expression in tumor tissue of the ARQ 092 and combination treated groups compared to the control group. This effect was expected as ARQ 092 inhibitor blocks AKT phosphorylation and prevents the inactive form from localizing into plasma membrane, protein levels of AKT are stable but AKT gene expression is decreased. Moreover, ARQ 092 and the combination treatment strongly downregulated the AKT-pathway downstream effector mTORC1 specifically in tumoral tissue while there is no significant difference in non-tumoral tissue. Ribosomal protein S6 kinase beta-1 (S6K1), another downstream effector of AKT and mTORC1, was significantly decreased by ARQ 092 and the combination in both tumoral and non-tumoral liver tissue.

Next, we studied whether sorafenib still inhibits the MAPK/ERK pathway or whether cells are already resistant to sorafenib. There was no difference in pERK/ERK ratio among all groups (Figure 4A and 4B) and MAPK1 mRNA levels were not altered among all groups (Figure 4C).

### Effect of treatment on immune system and tumor microenvironment

**Figure 5:**
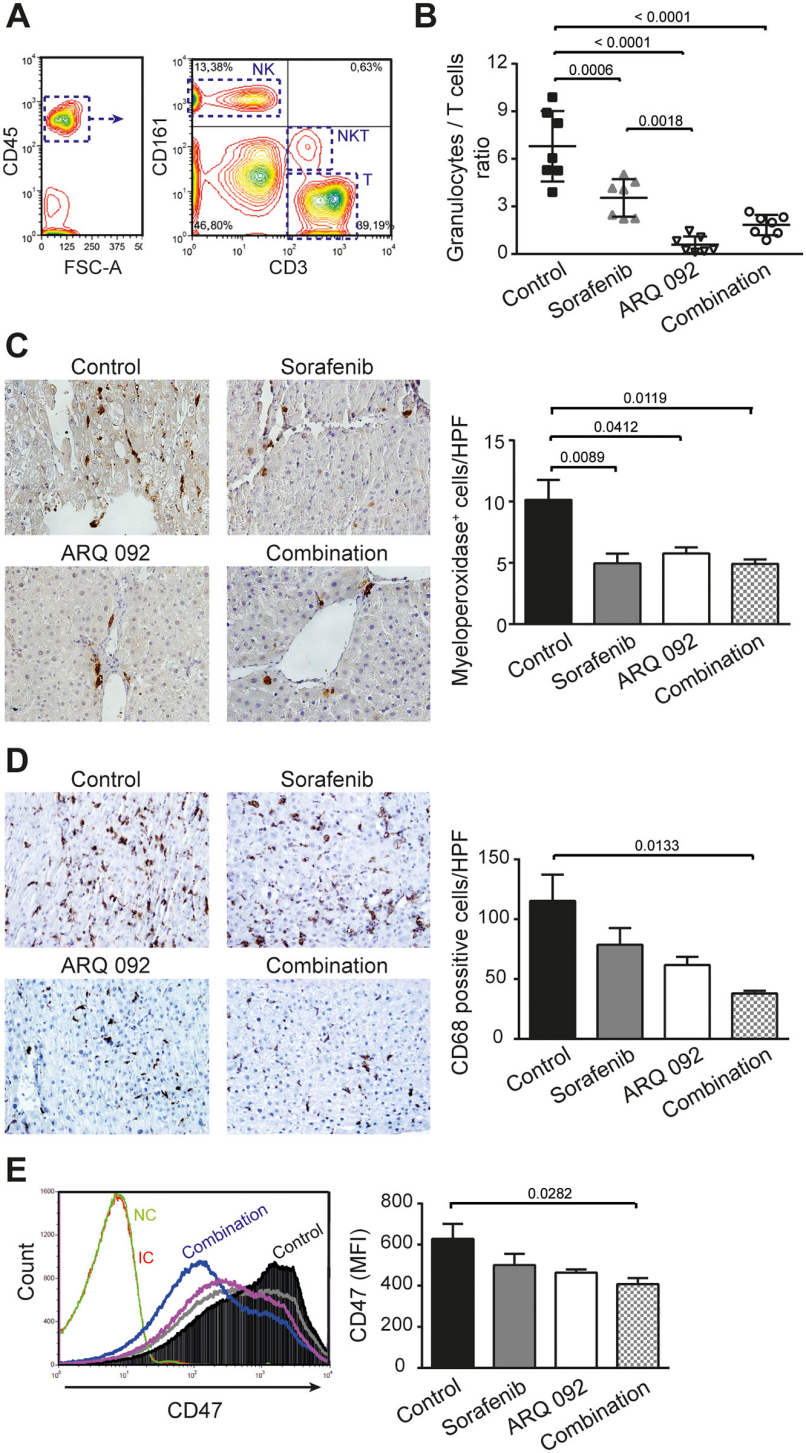
Effect of combination treatment on immune system and tumor microenvironment **(A)** Gating flow cytometry strategy to investigate immune cells. Lymphocytes were first identified according their FSC and SSC parameters and further gated based on their CD45^+^ expression. Among the CD45^+^ population, NK (CD161^high+^CD3^-^), NKT (CD161^low+^CD3^+^) and T (CD161^-^CD3^+^) cells were selected. **(B)** Granulocytes to lymphocytes ratio. Values are means ± SE. N=7/group. Comparison of means was done by ANOVA test with Tukey correction. **(C)** Representative histological images of livers stained with myeloperoxidase and the quantification of positive cells (neutrophils) per high power field (HPF). **(D)** Representative histological images of livers stained with CD68 and the quantification of positive cells (macrophages) per HPF. **(E)** Expression of CD47 in tumor liver tissue and quantification of mean fluorescence intensity (MFI) of CD47, sorafenib (grey line), ARQ 092 (purple line). Values are means ± SE. N=7/group. Comparison of means was done by ANOVA test with Tukey correction.

To characterize the effect of treatment on the immune system, whole fresh blood was analyzed by flow cytometry. Immune cells were identified based on CD45 expression and different populations of lymphocytes were then identified accordingly to their respective rat-specific markers: NK (CD161^high+^CD3^-^), NKT (CD161^low+^CD3^+^) and T (CD161^-^CD3^+^), Figure 5A. No difference in frequency of circulating NK or NKT cells was observed between groups (Supplementary Table 3). Interestingly, the frequency of T-cells in population of CD45^+^ was increased by ARQ 092 and the combination treatment compared to control and sorafenib. This effect was accompanied by a strong reduction in the number of circulating granulocytes (Supplementary Table 3), which together led to significant reduction of Granulocyte/T cell ratio, Figure 5B.

In liver tissue, flow cytometry analyses showed no differences in the population of T-cells, NK cells or NKT cells between experimental groups. Similarly, by immunohistochemistry, we observed no significant differences between groups in the frequency of intrahepatic CD3- and CD8-positive cells (data not shown).

In accordance with the decrease in granulocytes frequency in blood, we observed a significant decrease in accumulated neutrophils in the liver tissue of all treated groups compared to control, as determined by myeloperoxidase staining, Figure 5C. Another population of immune cells, which was significantly reduced in the tumor microenvironment of the treated animals compared to control, were macrophages. We observed significant reduction of CD68^+^ cells in rats treated by the combination treatment compared to control group, Figure 5D.

Tumor-initiating cells are characterized by high expression of CD47 [15]. Moreover, over-expression of CD47 is involved in sorafenib resistance [16]. Flow cytometry analyses revealed that highly expressing CD47 population in liver tissue was decreased by all treatments, with a significant effect in the combination group (Figure 5E).

All together, our data demonstrated that the combination treatment improves anti-tumor immune balance in the tumor itself and in blood, potentially explaining in part the anti-tumor effect of this combination. Moreover, the combination treatment significantly decreased a population of tumor-initiating cells.

## DISCUSSION

The heterogeneity of HCC is both genetic [17] and phenotypical/morphological [18], with the hallmarks of cancer exhibited in a complex manner such as localizations and times. This complex and multivariate tumor network that constantly responds to and influences liver environment is the main reason of the limited success of different targeted monotherapies tested in HCC [19]. Thus, combining multiple anti-cancer drugs seems to be a rational approach in the prevention of tumor resistance. Nonetheless, as this strategy usually induces a huge increase of toxicity, there is an urgent need to find well-tolerated and effective combinations of targeted therapy to treat HCC patients.

HCC is a hypervascularized tumor with an anarchic neoangiogenesis and is usually surrounded by a cirrhotic liver. These characteristics obviously influence drug metabolism, by making systemic drug delivery less effective and by leading to severe adverse events. Therefore, before testing the safety and efficacy of multitarget therapies in clinical trials, pre-clinical studies are essential and the most optimal animal model need to be chosen. Thus, to test the combination of sorafenib and the AKT inhibitor ARQ 092, we used a cirrhotic rat model with HCC that closely reproduce human HCC physiopathology. We observed that the combination of ARQ 092 with sorafenib additively reduced tumor progression and tumor size with a significant higher efficacy than sorafenib and ARQ 092 monotherapies. The anti-tumor effect was associated with a significant reduction of tumor cell proliferation and an increased apoptosis *in vivo*. Treatment of ARQ 092 showed marked reduction of tumor number similar to the combination treatment, whereas sorafenib only had a modest effect on tumor initiation. Similarly, the cell proliferation, determined by Ki67 staining, was strongly reduced exclusively by ARQ 092, suggesting that AKT inhibition may even block tumor initiation. To confirm this hypothesis in our animal model, further experiments with an earlier introduction of ARQ 092 (during the DEN-induction phase) are needed.

Sorafenib-ARQ 092 combination therapy was very successful not only in targeting the tumor, but also in amelioration of liver microenvironment. This is particularly important, because after HCC initiation, the tumor progression is finely regulated by tumor microenvironment which even later influences the tumor response to therapies. For instance, increased and irregular vasculature will allow small HCC lesions to progress and metastasize, which is a typical situation in the fibrotic liver characterised by constantly increased formation of blood vessels [20]. The mechanism of beneficial action of sorafenib on liver vascularisation was described previously [21]. Here, we showed that the combination of ARQ 092 and sorafenib improved the vascularization of liver tissue in an additive manner and additionally decreases expression of HIF-1 in tumor tissue. Similarly, the anti-fibrotic effect of sorafenib was clearly demonstrated by numerous experimental studies (reviewed in [22]). In our study, the anti-fibrotic effect of sorafenib was relatively modest. On the contrary, the sorafenib-ARQ 092 combination greatly shifted matrix regulatory pathway, leading to fibrosis resolution with a strong decrease of collagen accumulation. Another essential determinant of HCC progression and survival is cancer-associated inflammation, with TGFβ orchestrating a favorable microenvironment for tumor cell growth. Here we showed that expression of TGFβ in non-tumor tissue was downregulated in an additive way by sorafenib-ARQ 092 combination.

Recently, a meta-analysis showed that a high neutrophil-to-lymphocyte ratio indicates a poor prognosis in patients with HCC, representing a shift towards an increased pro-tumor inflammation and decreased anti-tumor immune functions [23, 24]. In our model, all treatments significantly decreased granulocyte-to-lymphocyte ratio compared to the control. The strong reduction of circulating neutrophils in rats treated by ARQ 092 monotherapy should to be taken into account. In fact, ARQ 092 was recently used to block neutrophils and decrease inflammation in sickle cell disease [25]. However, because neutrophils are the first responders to sites of acute injury and infection, the effect of ARQ 092 on circulating neutrophils needs to be mentioned.

The frequency of liver infiltrating neutrophils was similarly reduced by all treatments, while accumulation of macrophages was additively decreased by the sorafenib-ARQ 092 combination. This is particularly important as there is growing evidence of the key-role of neutrophils and macrophages in liver fibrosis and HCC progression [26–28]. On the other hand, we did not find significant changes in intrahepatic T-cells. However, we may not be able to study particular T-cell subpopulations due to specificity of antibodies against rat.

Despite difficulties induced by the presence of a cirrhosis in our DEN-rat model of HCC, the sorafenib-ARQ 092 combination showed enhanced efficacy with a good safety profile. The dose strategy 5 days on - 9 days off for ARQ 092 was based on a toxicity study (unpublished data) with very good tolerance. Similarly, to decrease sorafenib toxicity, the concentration of 10 mg/kg was used. In fact, our pilot experiments showed that in cirrhotic rats treated with sorafenib, a dose of 20 mg/kg causes severe adverse events including an important weight loss, demonstrating the complexity of HCC treatment and the importance of using an appropriate animal model to test HCC treatment efficacy and safety.

We identified a novel treatment choice for advanced HCC with cirrhotic background. The resutls from *in vitro* and *in vivo* studies clearly illustrated the significance of targeting AKT pathways that potentiates sorafenib treatment of HCC. The safety and efficacy of this combination strategy provides the possibility of improvement of therapeutic outcomes for advanced HCC patients.

## MATERIALS AND METHODS

### Cell lines and *in vitro* studies

In this study, we used three different human HCC cell lines (Hep3B, HuH7, and PLC/PRF/5) and one hepatoblastoma cell line (HepG2). While Hep3B is p53-depleted, HuH-7 and PLC/PRF/5 present p53 mutations and HepG2 is a wild-type p53-expressing cell line. No mutations in AKT were detected in mentioned cell lines (COSMIC database). Expression of p-AKT was reported to be normal in Hep3B and low in HepG2, HuH-7 and PLC/PRF/5 cell lines [4]. Culture conditions are described in supporting information.

A cell viability assay was performed by MTT (3-(4, 5-Dimethylthiazol-2-yl)-2, 5-diphenyltetrazolium bromide), apoptosis was assessed by flow cytometry analysis and cell migration was studied using a wound healing assay and by cell tracking with time-lapse microscopy as described in supporting information.

### Preparation of treatments

Preparation of ARQ 092 (ArQule Inc, USA), sorafenib (*in vitro* study: Bay 43-9006, Sigma-Aldrich, Germany; *in vivo* study: Nexavar^®^, Bayer HealthCare, Germany) and the combination treatment for *in vitro* and *in vivo* experiments is described in detail in the supporting information.

### Rat model and groups of treatment

Twenty-eight 6-week-old Fischer 344 male rats (Charles River Laboratories, France) were housed in the animal facility of Plateforme de Haute Technologie Animale (Jean Roget, University of Grenoble-Alpes, France). Rats were treated weekly with intra-peritoneal injections of 50 mg/kg of diethylnitrosamine (DEN) (Sigma-Aldrich, Germany), diluted in olive oil in order to obtain a fully developed HCC on a cirrhotic liver after 14 weeks [14]. Rats were randomized in 4 different groups (n=7/group) and treated during six weeks by i) sorafenib, ii) ARQ 092, iii) combination of ARQ 092 and sorafenib or iv) rested untreated (control), as specified in Supplementary Figure 1. ARQ 092 alone, sorafenib alone and combination (Sorafenib plus ARQ 092) treatments were dispensed by oral gavage for a period of six weeks. ARQ 092 treatment was administered 5 days on 9 days off, at the dose of 15 mg/kg/day for single treatment group same as for combination group, as recommended by the ArQule Inc. Sorafenib was administered continuously at the dose of 10 mg/kg/day for single treatment group as well as for the combination group. We used 10 mg/kg/day because igher concentrations of sorafenib were demonstrated to be toxic for cirrhotic rats ([11], pilot experiments and personal communication with Bayer AG).

Nutritional state was monitored by daily weighing of rats and protein-rich nutrition was added to the standard food in every cage where a loss of weight was observed. Food was withheld for 3-4 hours before animals were sacrificed.

All animals received humane care in accordance with Guidelines on the Humane Treatment of Laboratory Animals, and experiments were approved by the animal Ethic Committee: GIN Ethics Committee n°004.

### MRI studies

The imaging study was conducted on a 4.7 Tesla MR Imaging system (BioSpec 47/40 USR, Bruker Corporation, Germany). As illustrated in Supplementary Figure 1, all rats were subjected to 3 MRI scans: MRI1 was performed before randomization, MRI2 was performed after 3 weeks of treatment and MRI3 after 6 weeks of treatment. The protocol for image acquisition and analysis is detailed in supporting information. MRI analysis was done by an investigator who was blinded of treatment allocation.

### Histopathological, immunohistochemical and immunofluorescence analyses

After the third MRI scan, all rats were euthanized with vena cava blood sampling for haematologic and biochemical analyses. Each liver was weighed, the diameter of the five largest tumors was measured and the number of tumors larger than 1 mm on the surface of the liver was counted, all in a blinded manner. Tumor proliferation and apoptosis were studied by using anti-Ki67 antibody and TUNEL marker. Tissue vascularization was determined by CD34 immunostaining. Histological analysis of fibrosis was performed by sirius red-staining of collagen. Analyses were performed in collaboration with experienced pathologist (CHU-Grenoble Département d’Anatomie et de Cytologie Pathologiques). Protocols are described in supporting information.

Serum and plasma were tested for liver and kidney safety markers (albumin, ALP, ALT, AST, prothrombin time, total bilirubin, cholesterol, GGT, glucose, creatinine, Table 1) by Charles River Clinical pathology Services using Olympus and Stago instruments. Liver triglycerides were measured as described previously [11].

### Pathways analysis

Western blot analysis of pAKT^(Ser473)^/AKT and pERK/ERK, and real-time polymerase chain reaction (qPCR) analyses were performed on tumor and non-tumor tissues. Protocols are described in the supporting information.

### Flow cytometric analysis

Cells were recovered from liver tissue by mechanical disruption as described previously [29] and whole blood samples were used in case of blood analyses. Cells without any stimulation were immune-stained for flow cytometric analysis. The protocol is described in the supporting information.

### Statistical analysis

All comparisons of means were calculated by using ANOVA tests with Tukey HSD correction for multiple means comparisons, and independent T-tests only when two means were compared. Data are presented as mean values ± standard error mean (SEM). Statistical analyses were performed using Prism 6 (GraphPad Software Inc., CA, USA).

## SUPPLEMENTARY MATERIALS FIGURES AND TABLES



## Abbreviations

HCC: Hepatocellular carcinoma
VEGF-R: vascular endothelial growth factor receptor
PDGF-R: platelet-derived growth factor receptor
PI3K: phosphatidylinositol-3 kinases
AKT: Protein kinase B, PKB
mTOR: mechanistic target of rapamycin
mTORC1: mTOR complex 1
MTT: 3-(4, 5-dimethylthiazolyl-2)-2, 5-diphenyltetrazolium bromide
DEN: diethylnitrosamine
MRI: Magnetic Resonance Imaging
pAKT: phosphorylated AKT
ERK: extracellular-signal-regulated kinase
pERK: phosphorylated ERK
qPCR: real-time polymerase chain reaction
SEM: standard error mean
IC50: inhibitory concentration 50
CI: combination index
ALP: Alkaline Phosphatase
GGT: Gamma glutamyl transpeptidase
AFP: Alpha fetoprotein
HIF-1: hypoxia-inducible factor
α-SMA: alpha smooth muscle actin
TGFβ1: Transforming growth factor β1
TIMP-1: tissue inhibitor of metalloproteinases-1
MMP: matrix metalloproteinase
S6K1: Ribosomal protein S6 kinase
