# Supplementary material for: Combination of AKT inhibitor ARQ 092 and sorafenib potentiates inhibition of tumor progression in cirrhotic rat model of hepatocellular carcinoma
Source: Oncotarget. 2018 Jan 23;9(13):11145–58. doi: 10.18632/oncotarget.24298 (PMC5834253; doi:10.18632/oncotarget.24298)
Supplement: Supplementary file 1 [file oncotarget-09-11145-s001.pdf]

Combination of AKT inhibitor ARQ 092 and sorafenib potentiates inhibition of tumor progression in cirrhotic rat model of hepatocellular carcinoma

SUPPLEMENTARY MATERIALS

MATERIALS AND METHODS

Cell lines

HepG2 and Hep3B cells were cultured in Minimum Essential Medium (MEM, GIBCO™, Life Technologies, UK), with GlutaMAX™ Supplement. Huh-7 cells were incubated in Dulbecco's Modified Eagle Medium (DMEM, GIBCO™, Life technologies), with high glucose, and GlutaMAX™ supplement. PLC/PRF/5 cells were cultured in DMEM supplemented with 1% of sodium pyruvate (GIBCO™, Life technologies). Mediums were supplemented with 10% of Fetal Bovine Serum and 1% of antibiotics (Pen Strep, Life Technologies).

Preparation of treatments

ARQ 092 was kindly provided by ArQule Inc (Woburn, MA, USA).

For *in vitro* studies, Sorafenib tosylate (Bay 43-9006, Sigma-Aldrich, Germany), and ARQ 092 were dissolved in pure dimethylsulfoxyde (DMSO, Sigma-Aldrich). ARQ 092 was stored at -20°C temperature and protected from light. Combination was prepared by mixing of single drugs just before the start of *in vitro* experiments.

For *in vivo* study, 200 mg Sorafenib tosylate tablets (Nexavar®, Bayer HealthCare, Germany) were used and prepared as previously described (1). The sugar coating was first dissolved in DMSO and Sorafenib was mixed with 1 mL of poly-oxyl castor oil (Cremophor® EL, Sigma-Aldrich) and 1 mL of 95% ethanol per tablet to emulsify and solubilize it. To finish, the emulsion was diluted in purified water to obtain a 10 mg/mL solution of Sorafenib suitable for oral gavage.

ARQ 092 was dissolved in a 0.01M phosphoric acid solution to obtain a 7.5 mg/mL ARQ 092 solution suitable for oral gavage with a final pH of 2.25 ± 0.15.

Fresh solutions of each drug were prepared every week and stored at room temperature, protected from light. Combination was prepared by mixing the same volume of single drugs just before oral gavage.

Cell viability assay

Cell viability was determined by MTT (Sigma-Aldrich) (3-(4, 5-Dimethylthiazol-2-yl)-2, 5-diphenyltetrazolium bromide) assay in 4 cell lines. IC20 and IC50 values of single treatments (Sorafenib, ARQ 092) were determined previously. To determine Combination Index (CI) values, different concentrations of same IC50:IC50 ratio values of Sorafenib and ARQ 092 were used. For Combination treatment, cells were exposed to 11 different concentrations ($IC_{50}^{Sorafenib}+IC_{50}^{ARQ\ 092}/1000$, $IC_{50}^{Sorafenib}+IC_{50}^{ARQ\ 092}/500$, $IC_{50}^{Sorafenib}+IC_{50}^{ARQ\ 092}/200$, $IC_{50}^{Sorafenib}+IC_{50}^{ARQ\ 092}/100$, $IC_{50}^{Sorafenib}+IC_{50}^{ARQ\ 092}/50$, $IC_{50}^{Sorafenib}+IC_{50}^{ARQ\ 092}/20$, $IC_{50}^{Sorafenib}+IC_{50}^{ARQ\ 092}/10$, $IC_{50}^{Sorafenib}+IC_{50}^{ARQ\ 092}/5$, IC50/2, 1x $IC_{50}^{Sorafenib}+IC_{50}^{ARQ\ 092}$, 2x $IC_{50}^{Sorafenib}+IC_{50}^{ARQ\ 092}$) during 48h. CI values were calculated with an implementation of the Median-Effect approach of Chouand Talalay using CompuSyn software as described previously (2). CI=1 indicates an additive effect, CI>1 indicates antagonism, CI<1 indicates a synergistic effect and CI <0.1 indicates very strong synergistic effect (3).

As a negative control, cells were incubated in the same medium with 1 % DMSO without drug. Each sample was analyzed on three replicates and all experiments were repeated three times.

Apoptosis assay

Apoptosis assay was performed on 4 cell lines treated with IC20 and IC50 concentrations of Sorafenib, ARQ092 or with Combination IC50/200 and Combination IC50/10 during 48h. Annexin V (BD biosciences, USA; early-stage apoptosis marker) conjugated to FITC and 7-amino-actinomycin D (7-AAD, BD biosciences) positive cells were measured by flow cytometry (BD Accuri C6, BD biosciences, USA).

Cell migration

Cell migration was assessed on 4 cell lines by wound-healing assay and cells were then treated with IC20 and IC50 concentrations of Sorafenib or ARQ 092 or with Combination IC50/200 and Combination IC50/10. Images were captured every hour by time-lapse microscopy at 37°C, 5% CO_2 with Zeiss AxioVert 100M connected to a MicroMAX B/W (6.7x6.7 µm, -15°, ~ 3 im/s) camera using MetaMorph ® software (MetaMorph Inc., USA). The width of the wound was quantified at 24 h by software ImageJ. Data are presented as relative percentage of closed-wound. The experiments were

performed in duplicates and repeated at least three times. Cell velocity was determined via tracking cells by time-lapse microscopy. Cells were then treated with IC20 and IC50 concentrations of ARQ 092 or sorafenib and images of cells were captured every hour by time-lapse microscopy with Zeiss AxioVert 100M connected to a MicroMAX B/W camera. To estimate the velocity, 4 different areas per sample were captured and 5 cells per area were tracked using the Manual Tracking plugin for Image J.

MRI studies

Imaging study was performed with a 4.7 Tesla MR Imaging system (BioSpec 47/40 USR, Bruker Corporation, Germany) and Transmit/Receive Volume Array Coil for rat body 8x2 (Bruker Corporation, Germany) in the Grenoble MRI facility IRMaGE. Rats were fitted in ventral decubitus position and anesthetized with isoflurane inhalation (Forane®, Abbott, USA). Breathing was continuously monitored to maintain a respiratory rate between 35 and 45 breaths per minute and body temperature was maintained around 37°C.

We used Turbo rapid acquisition with relaxation enhancement T2-weighted (Turbo-RARE T2) sequence (repetition time (TR): 1532.9 msec, echo time (TE): 27.4 msec, flip angle (FA): 180°) with a field of view (FOV) of 55 x 55 mm, 20 slices, a thickness and a slice separation of 2 mm, and were realized with a respiratory triggered acquisition to reduce artefacts. MRI parameters adjustment and image acquisition were realized by using Paravision 5.1 software.

A morphological analysis was realized based on the TurboRARE T2 sequences and according to the Response Evaluation Criteria in Solid Tumors (RECIST) criteria. Ten liver tumors were selected and measured on MRI1, 2 and 3. Estimated tumor size corresponded to the sum of the largest diameter of these 10 lesions. For each rat, MRI1 was considered as the baseline (i.e.: 0%) and tumor progression corresponded to the comparison between MRI2 or 3 and the baseline, (i.e: "(tumor $size_{MRI2/3}$ - tumor $size_{MRI1}$)/tumor $size_{MRI1}$).

Histopathological, immunohistochemical and immunofluorescence analyses

Liver tissues were fixed in 10% formalin solution, neutral buffered (Sigma-Aldrich). Paraffin-embedded, four-micrometer sections were then stained with Hematoxylin/Eosin.

To detect proliferating cells, paraffin-embedded sections were incubated overnight at 4°C with the primary anti-Ki67 antibody (Rabbit, clone SP6, Thermofisher scientific, USA), followed by incubation with the peroxidase-conjugated bovine anti-rabbit IgG (Jackson ImmunoResearch, USA). DAB was used as the chromogen for Ki67 immunodetection. For $Ki67^+$ cells, data are presented as positive cell nuclei per area (high-power fields; 20x magnification).

Apoptotic cells were analysed by ApoBrdU-IHC DNA Fragmentation Assay Kit (Biovision, USA) and methyl green solution was used for counter staining the cells. Data are presented as apoptotic cells per area (high-power fields; 20x magnification).

To detect vascularisation, paraffin-embedded sections were blocked by 10% donkey serum and then incubated overnight at 4°C with anti-rat CD34 antibody (Goat, AF4117, R&D Systems; Minneapolis, USA), followed by incubation with Alexa 647-conjugated donkey anti-goat IgG (Life Technologies, Carlsbad, CA, USA). Images were captured using ApoTome microscope (Carl Zeiss, Germany) equipped with a camera AxioCam MRm and collected by AxioVision software. Positive area was quantified using ImageJ software (NIH, USA) on 15 randomly selected fields/section (10x magnification).

Collagen was detected on paraffin-embedded sections with picro-sirius red stain solution (Sigma-Aldrich) and staining was subsequently quantified by MetaMorph ® software in 10 randomly selected fields/section (10x magnification).

Oil Red O staining was performed on 7µm cryosections, prepared from formalin pre-fixed liver samples. Sections were stained with freshly prepared Oil Red O in isopropanol. Oil Red O staining provides chromogenic as well as fluorescent signals, therefore we used red channel to detect staining. Images were captured using ApoTome microscope (Carl Zeiss, Germany) equipped with a camera AxioCam MRm, collected by AxioVision software and quantified using ImageJ software (NIH, USA). For Oil Red O^+ liver area, data are presented as Oil Red O positive area in percent of total tissue area. Six random areas per each liver section were analysed.

Real-time polymerase chain reaction (qPCR)

Total RNA was extracted from frozen rat liver tissue samples. RNA purification was performed with RNeasy Mini Kit ® (Qiagen, USA). Reverse transcription was realized with Transcriptor First Strand cDNA Synthesis Kit ® (Life science, Roche), and amplification reactions were performed in a total volume of 20µL by using a Thermocycler sequence detector (BioRad CFX96, USA) with qPCR kit Mesa Green qPCR MasterMix Plus for SYBR Assay ® (Eurogentec, Belgium).

GADPH was used as housekeeping gene. Primers were designed with Primer 3 software (version 4.0.0) and verified on BLAST. Oligonucleotide sequences were synthetized by Eurofins Genomics ® in 0.01µmol scale, with a Salt Free level of purification. Every analysis was done in duplicates.

Immunoblot analysis

Liver homogenates were prepared in RIPA buffer (50 mM Tris; 1% NP40; 0.5% deoxycholic acid sodium salt; 150 mM NaCl; 1 mM EGTA) containing Protease and Phosphatase Inhibitors, and proteins were quantified with NanoDrop ® (Thermofisher scientific). Proteins were then denatured in Laemmli Sample Buffer (Bio-Rad) containing 5% β-mercaptoethanol and separated by gel electrophoresis (Mini Protean Gels ®, Bio-Rad) and transferred to polyvinylidene difluoride (PVDF; Bio-Rad) membranes using a wet blot method. Membranes were blocked in TBS-Tween solution with 5% BSA for 1 h at 4°C. Primary antibodies against p-Akt $^{(Ser473)}$, Akt $^{(pan)}$, pERK $^{(Thr202/Tyr204)}$, ERK $^{(p44/42\ MAPK)}$, β-actin (all Cell Signaling Technology, USA) were incubated at 4°C overnight under shaking conditions. Incubation with the secondary antibody (HRP-anti rabbit IgG, 1:2000; Cell Signaling) was performed under shaking conditions for 1 h. Detection was achieved with Clarity™ Western ECL Blotting Substrate (Bio-Rad) using a ChemiDoc™ MP Imaging System (Bio-Rad). Densitometric quantification of the bands was performed using the Image Lab™ Software (Bio-Rad).

Flow cytometric analysis

Cells were recovered from liver tissue by mechanical disruption and whole blood samples were used in case of blood analyses. Cells were immunostained for flow cytometric analysis without any stimulation. Following anti-rat antibodies were used: CD45 (Clone:OX-1, APC/Cy7, BioLegend), CD3 (Clone:1F4, BV605, BD Horizont), Granulocytes (Clone:RP-1, PE, BD Pharmingen), CD8 (Clone:G28, APC, BioLegend), CD4 (Clone:W3/25, PE/Cy7, BioLegend), CD161 (Clone:3.2.3, FITC, BioLegend), CD90 (Clone:OX-7, BV711, BioLegend), CD47 (PE, OX-101, BioLegend). Nonviable cells were stained by Zombie UV™ Fixable Viability Kit (BioLegend) and excluded from the analysis. Isotype-matched antibodies were used as control. Data were acquired on BD-LSRII flow cytometer (BD Biosciences, Le Pont-De-Claix, France), collected with BD FACSDiva 6.3.1 software and analyzed using FCS Express 6 PLUS software.

REFERENCES

1. Liu L, Cao Y, Chen C, Zhang X, McNabola A, Wilkie D, Wilhelm S, Lynch M, Carter C. Sorafenib blocks the RAF/MEK/ERK pathway, inhibits tumor angiogenesis, and induces tumor cell apoptosis in hepatocellular carcinoma model PLC/PRF/5. Cancer Res. 2006; 66:11851–58.
2. Chou TC. Theoretical basis, experimental design, and computerized simulation of synergism and antagonism in drug combination studies. Pharmacol Rev. 2006; 58:621–81.
3. Foucquier J, Guedj M. Analysis of drug combinations: current methodological landscape. Pharmacol Res Perspect. 2015; 3:e00149.

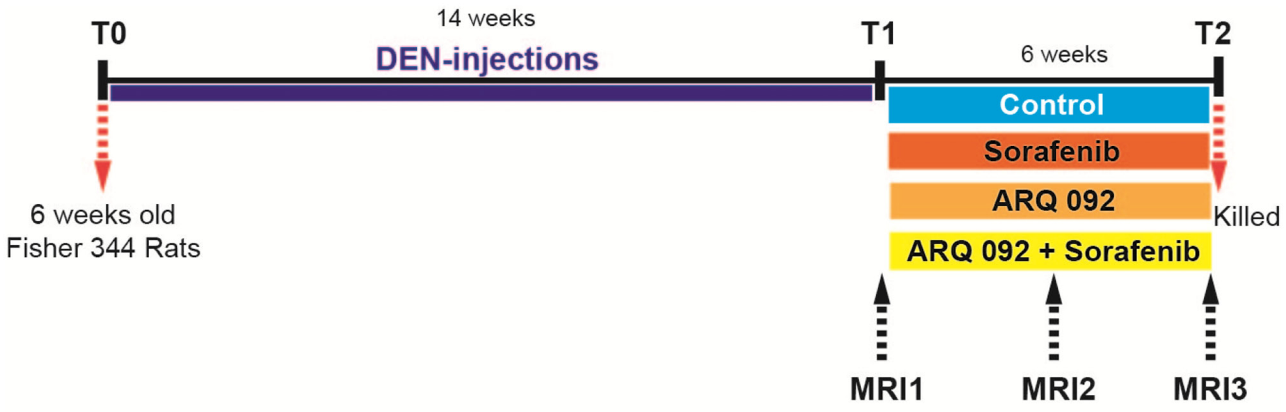

Supplementary Figure 1: Timeline of study. To characterise the efficacy of combination treatment of sorafenib and AKT inhibitor ARQ 092 on HCC, 6 weeks old Fischer 344 male rats were treated weekly with intra-peritoneal injections of 50 mg/kg diethylnitrosamine (DEN) during 14 weeks. 20-weeks old DEN-induced cirrhotic rats with HCC were treated during six weeks by sorafenib, ARQ 092, combination of ARQ 092 and sorafenib or rested untreated (Control), n=7/group.

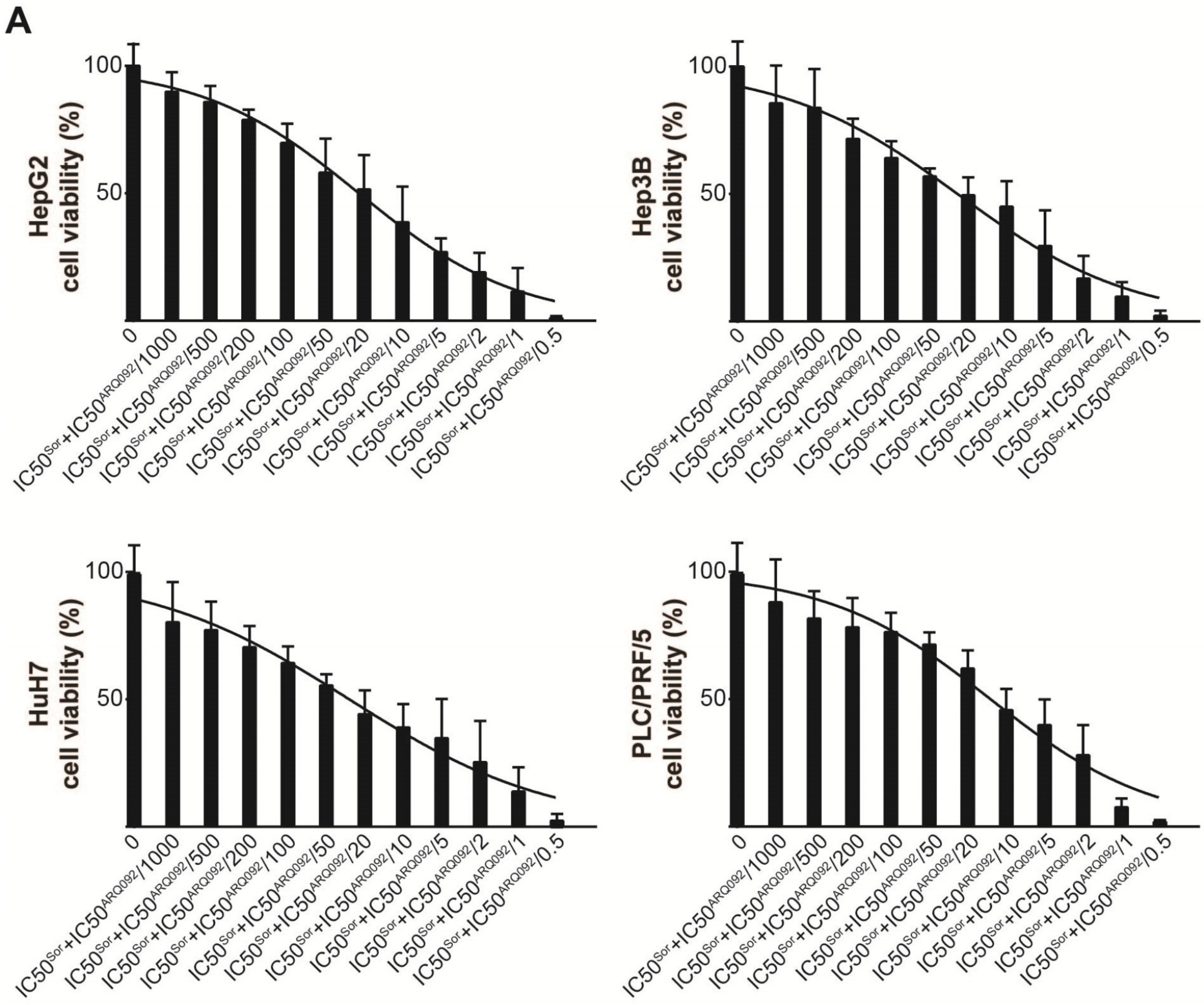

Supplementary Figure 2: Effects of combination treatment on cell growth. (A) MTT assay on HepG2 (upper left), Hep3B (upper right), HuH7 (lower left) and PLC/PRF/5 (lower right) cell lines after 48h of treatments showing significant decrease in cell viability with increasing concentrations of combination treatment of $IC_{50}^{Sorafenib}$+$IC_{50}^{ARQ\ 092}$. Values are expressed as the mean ± SEM of three independent experiments performed in triplicate.

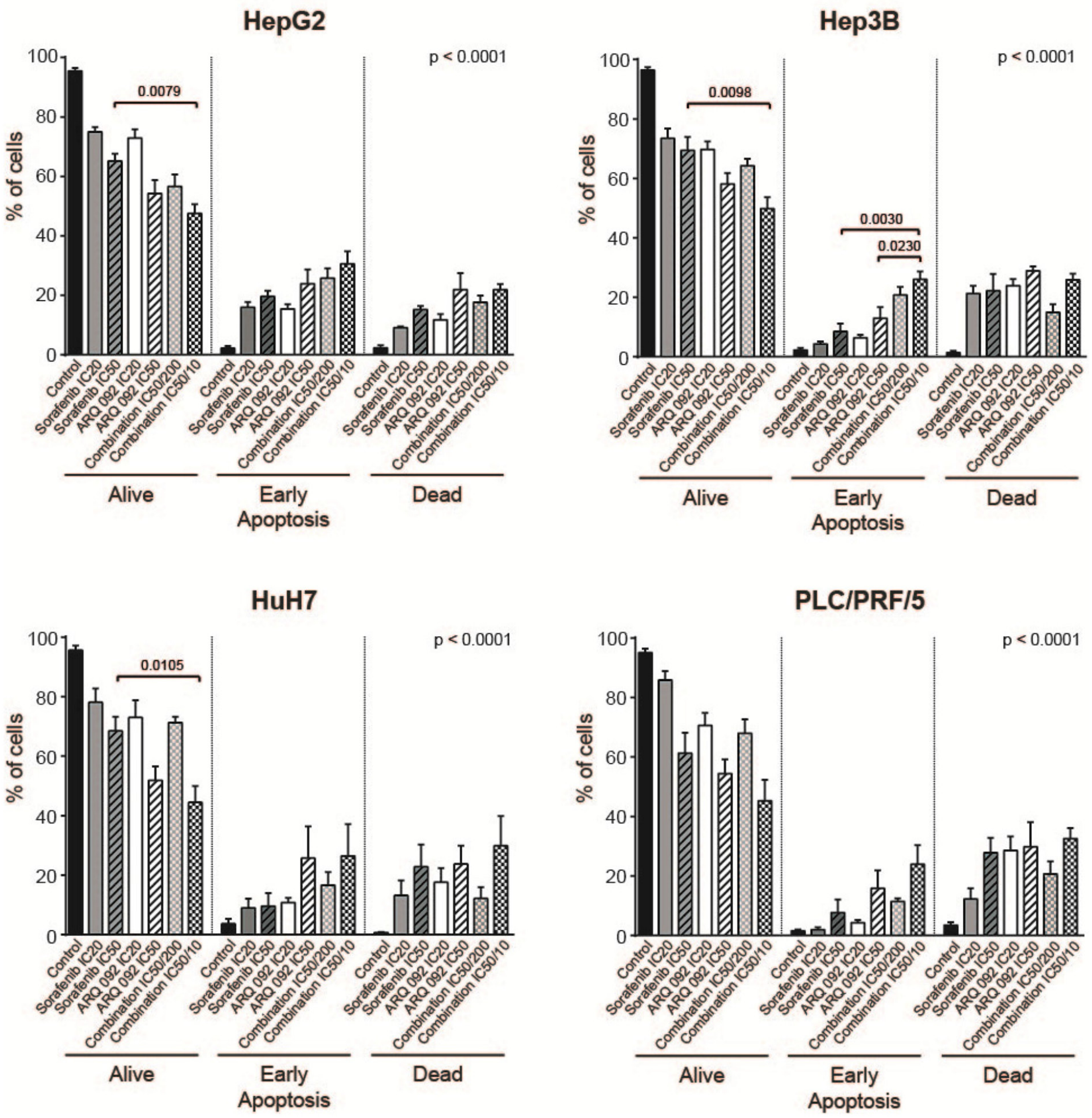

Supplementary Figure 3: Effects of combination treatment on apoptosis. HepG2 (upper left), Hep3B (upper right), HuH7 (lower left) and PLC/PRF/5 (lower right) cell lines after 48h of exposure to treatments. Values are expressed as means ± SEM of three independent experiments performed in duplicates. P values in graph represent ANOVA comparison of ARQ 092 IC50, Sorafenib IC50 and Combination IC50/10. P value of ANOVA test of all groups is indicated in the corner of the graph.

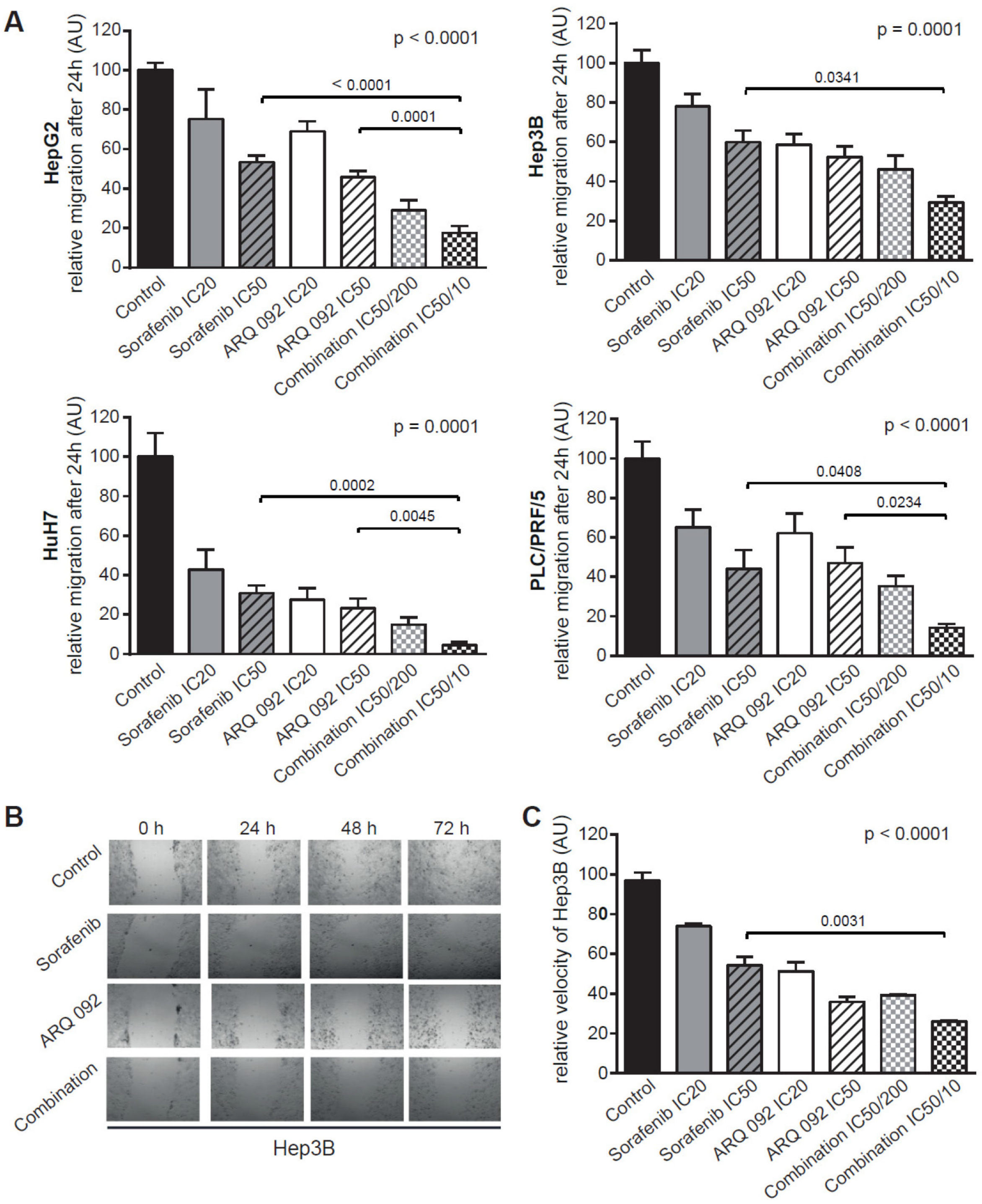

Supplementary Figure 4: Effect of Combination treatment on migration of HCC cell lines. **(A)** Quantification of cell migration studied by wound healing assay (decrease of width of the wound after 24h) in HepG2 (upper left), Hep3B (upper right), HuH7 (lower left) and PLC/PRF/5 (lower right) cell lines. **(B)** Representative pictures of wound healing assay in Hep3B cell line. **(C)** Quantification of cell velocity determined by cell tracking with time-lapse microscopy Control was set as 100%, values are means ± SE from three independent experiments performed in triplicates in duplicates. P values in graph represent ANOVA comparison of ARQ 092 IC50, Sorafenib IC50 and Combination IC50/10. P value of ANOVA test of all groups is indicated in the corner of the graph.

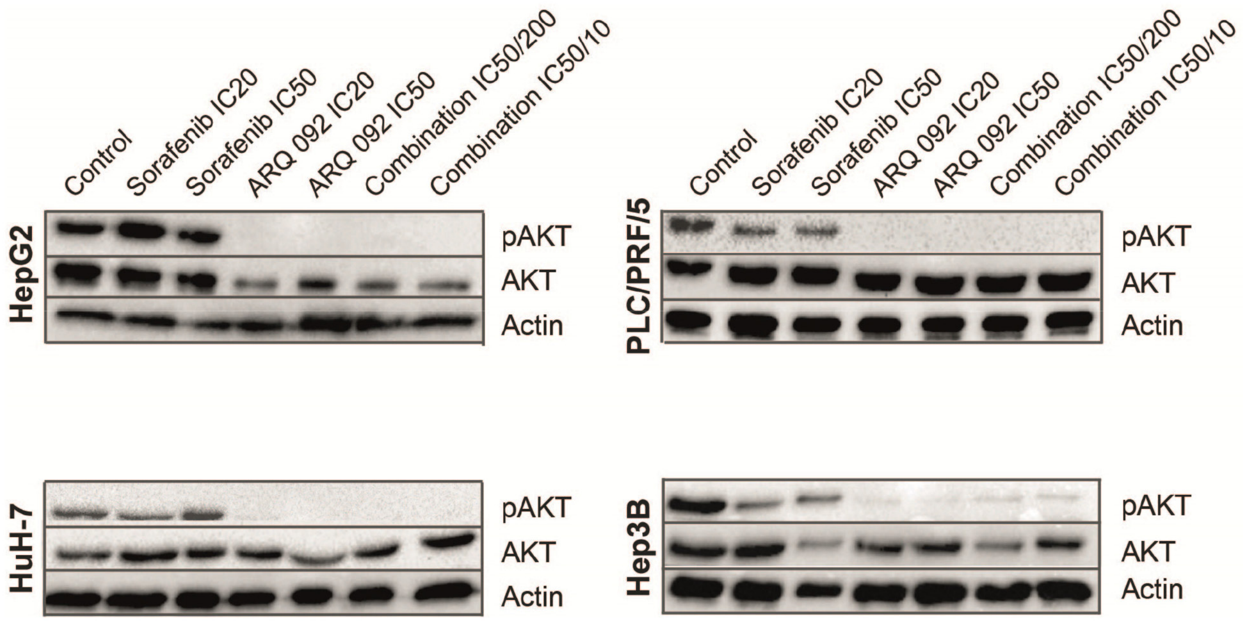

Supplementary Figure 5: Effect of combination treatment on AKT pathway in human cell lines. Western blot analysis of pAK/AKT and Actin in HepG2 (upper left), Hep3B (upper right), HuH7 (lower left) and PLC/PRF/5 (lower right) cell lines after 2 h exposure to treatment. Similar results were obtained after 24 h and 48 h of exposure - data not shown.

Supplementary Table 1: Half maximal inhibitory concentration (IC50) values

		Sorafenib	ARQ 092	p-value
		(μM)		
HepG2	IC50	10.5 ±0.04	3.7 ±0.03	0.0019
Hep3B	IC50	6.7 ±0.03	1.2 ±0.06	< 0.0001
HuH7	IC50	11.6 ±0.03	2.9 ±0.08	0.0027
PLC/PRF/5	IC50	12.7 ±0.04	7.0 ±0.08	0.0830

IC50 values of ARQ 092 and sorafenib in Hep3B, Huh-7, HepG2 and PLC/PRF/5 cell lines after 48h of exposure. Values are expressed as the mean ± SEM of three independent experiments performed in triplicate. Comparison of means were done with T-test.

Supplementary Table 2: Combination indexes

Cell line	$IC_{50}^{Sorafenib}$: $IC_{50}^{ARQ\ 092}$ (µM)	CI values (ED50)
HepG2	11:4	0.072
Hep3B	7:1	0.053
HuH7	12:3	0.054
PLC/PRF/5	13:7	0.085

Table of $IC_{50}^{Sorafenib}$: $IC_{50}^{ARQ\ 092}$ concentrations and calculated Combination indexes (CI) at effective dose 50 (ED50). CI=1 indicates an additive effect, CI>1 indicates antagonism, CI<1 indicates a synergistic effect and CI <0.1 indicates very strong synergistic effect (3).

Supplementary Table 3: Effect on circulating T cells, NK cells, NKT cells and Granulocytes

		Control (n=7)	Sorafenib (n=7)	ARQ 092 (n=7)	Combination (n=7)	ANOVA p-values
**T cells**	% of $CD3^{+}CD161^{-}$ cells in $CD45^{+}$ population	32.9±3.6	34.5±4.1	66.7±3.6****, $^{\#\#\#\#}$	54.2±2.9**,$^{\#\#}$	*<0.0001*
	% in living cells	2.0±0.5	5.7±1.3	7.6±1.8*	4.7±1.4	*0.0481*
**NK cells**	% of $CD3^{-}CD161^{high+}$ cells in $CD45^{+}$ population	8.9±2.0	9.6±2.2	7.4±1.4	10.4±2.0	*0.7279*
**NKT cells**	% of $CD3^{+}CD161^{low+}$ cells in $CD45^{+}$ population	4.0±0.5	4.5±0.4	3.5±0.3	4.5±0.4	*0.2689*
**Granulocytes**	% in living cells	13.3±3.1	16.1±2.0	1.5±0.2**,$^{\#\#\#}$	5.4±1.0*,$^{\#\#}$	*<0.0001*

Values are means ± SE. Significant difference compared to control; *: p<0.05; **: p<0.01; ***: p<0.001; ****: p<0.0001. Significant difference between ARQ 092 and Sorafenib; $^{\#\#}$: p<0.01; $^{\#\#\#}$: p<0.001; $^{\#\#\#\#}$: p<0.0001.
